# Postoperative outcomes after conventional, robotic-arm-assisted, and pressure-sensor-guided cruciate-retaining total knee arthroplasty: a retrospective cohort study

**DOI:** 10.1007/s11701-026-03954-w

**Published:** 2026-09-15

**Authors:** Tsz-Lung Choi, Gloria Yan-Ting Lam, Xueyou Zhang, Mingde Cao, Rex Wang-Fung Mak, Dennis Cham-Kit Wong, Patrick Shu-Hang Yung, Michael Tim-Yun Ong

**Affiliations:** 1 Department of Orthopaedics and Traumatology, Alice Ho Miu Ling Nethersole Hospital, Hong Kong SAR, China; 2 Department of Orthopaedics and Traumatology, Faculty of Medicine, The Chinese University of Hong Kong, Hong Kong SAR, China; 3 Department of Orthopaedics and Traumatology, Prince of Wales Hospital, Hong Kong SAR, China

**Keywords:** Total knee arthroplasty, Cruciate-retaining, Robotic-arm-assisted surgery, MAKO, Intraoperative pressure sensor, Verasense

## Abstract

Conventional cruciate-retaining total knee arthroplasty (CR-TKA) uses manual instrumentation and surgeon-directed soft-tissue assessment, whereas MAKO robotic-arm assistance adds geometry- and gap-distance guidance and Verasense pressure sensing adds compartment-load feedback. Whether these approaches are associated with different clinically relevant postoperative outcomes remains uncertain. To evaluate associations between surgical technique and postoperative pain, range of motion, and function across conventional, MAKO-assisted, and Verasense-guided CR-TKA, and explore whether preoperative severity modified associations with postoperative outcomes. The single-centre cohort included 173 knees in 139 patients (36 Conventional, 92 MAKO, 45 Verasense). Analyses proceeded from post hoc favourable change to principal covariate-adjusted postoperative outcomes, followed by post hoc baseline-severity interactions. Principal models adjusted for baseline value, age, sex, and body mass index. Knee-level outcomes accounted for within-patient clustering; the Knee Society Function Score was analysed once per patient. Holm correction was applied within each stated test family. No outcome showed a Holm-adjusted group difference in favourable improvement or adjusted postoperative status. Verasense versus MAKO showed greater pain improvement (0.769 points; Holm P = 0.2195) and lower adjusted postoperative pain (−0.318 points; Holm P = 0.2546), but neither met the corrected criterion. Postoperative pain was zero in 125 of 149 knees (83.9%). The Knee Society Function Score severity interaction disappeared within the baseline range shared by all groups. We found no robust evidence that conventional instrumentation, geometry-based MAKO assistance, and load-based Verasense guidance were associated with different postoperative outcomes or that preoperative severity showed a reproducible technique-dependent association with outcomes. The Verasense-versus-MAKO pain pattern was hypothesis-generating and established neither superiority nor equivalence.

**Supplementary Information:**

The online version contains supplementary material available at https://doi.org/10.1007/s11701-026-03954-w.

## Introduction

Appropriate soft-tissue balance is central to cruciate-retaining total knee arthroplasty (CR-TKA), because the retained posterior cruciate ligament contributes to stability throughout flexion [1, 2]. Balancing remains technically demanding: ligament quality and tension vary among osteoarthritic knees, and conventional assessment combines spacer blocks, manual stress testing, and surgeon judgement [3]. These methods are clinically familiar, but uncertainty remains in both the geometry of the reconstructed knee and the loads transmitted across its compartments.

MAKO and Verasense provide different forms of intraoperative information rather than interchangeable forms of assistance. MAKO combines computed-tomography-based planning and haptically constrained bone-cut execution with geometry-based information on alignment, component position, and medial and lateral joint-gap distances [4–9]. Verasense directly measures medial and lateral compartment loads during trialling, providing load-based feedback for balancing adjustments after bone preparation [9–17]. Thus, MAKO primarily characterises and controls reconstructed geometry, whereas Verasense quantifies how loads are distributed across that reconstruction.

The clinical relevance of adding robotic or sensor guidance to conventional TKA depends on whether gains in technical control are reflected in outcomes that patients notice. In the RACER-Knee pragmatic multicentre randomised trial, MAKO did not provide a clinically meaningful 12-month Forgotten Joint Score benefit over conventional TKA [18]. A recent meta-analysis of randomised trials found more consistent improvements in alignment accuracy than in WOMAC or Oxford Knee Score outcomes [19], while recent systematic reviews found no consistent functional or range-of-motion advantage from sensor-guided balancing [10, 12–17, 20–25]. Direct evidence comparing Conventional, MAKO, and Verasense as separate approaches remains limited. The clinically relevant question is whether these distinct forms of intraoperative guidance are associated with meaningful differences in postoperative pain, range of motion, and function.

We therefore examined whether the three surgical approaches were associated with differences in postoperative VAS, range of motion, and function. Three questions were examined in the order reported below. First, a post hoc clinically intuitive analysis compared observed pre-to-postoperative improvement among techniques. Second, the principal adjusted analysis compared postoperative outcomes after accounting for the corresponding baseline outcome, age, sex, and body mass index (BMI). Third, a post hoc hypothesis-generating analysis examined whether preoperative severity modified relative outcomes across techniques.

## Materials and methods

### Study design and ethics

This single-centre retrospective cohort study was approved by the Joint Chinese University of Hong Kong–New Territories East Cluster Clinical Research Ethics Committee (Ref. 2022.637), and individual informed consent was waived because retrospective, de-identified data were analysed. The study was conducted in accordance with the Declaration of Helsinki and is reported in accordance with the STROBE statement [26].

Clinical, procedural, and outcome data were obtained from an institutional dataset. All records within that dataset that met the stated eligibility criteria during the study period were included.

The overall procedure period extended from 6 August 2019 to 29 August 2022, and postoperative follow-up records extended through 4 May 2023. Conventional procedures occurred intermittently from 6 August 2019 to 29 August 2022, MAKO procedures from 5 January 2021 to 18 January 2022, and Verasense procedures from 22 March to 26 August 2022. These ranges represent the observed dates of included procedures rather than formal institutional implementation periods.

### Cohort definition

Eligible records were primary CR-TKAs for osteoarthritis using the Triathlon CR system and carrying an exact Conventional, MAKO, or Verasense technique label. The cohort was locked before the current statistical modelling, including 173 knees in 139 patients. Thirty-four patients contributed two knees; both knees were treated with the same technique in every bilateral patient. Missing postoperative outcomes did not exclude an otherwise eligible knee; each endpoint used its own paired complete-case sample.

Procedures were performed by three fellowship-trained arthroplasty surgeons, each of whom contributed cases to all three technique groups. Technique was not assigned by randomisation or a formal patient-level protocol. Within the observed treatment periods, selection reflected routine clinical practice and technology availability.

All groups used the same CR implant family and followed a standard institutional perioperative pathway, including spinal anaesthesia, tranexamic acid, multimodal analgesia, and standardised inpatient and outpatient rehabilitation.

Conventional (36 knees): Targeted mechanical alignment using manual femoral and tibial instrumentation and standard manual soft-tissue balancing.

MAKO (92 knees): Targeted restricted kinematic alignment using computed-tomography-based planning and haptically guided robotic-arm bone preparation. Soft-tissue balancing involved applying manual stress to allow the robotic system to record the joint gap, with implant positioning subsequently adjusted based on these dynamic recordings.

Verasense (45 knees): Targeted mechanical alignment using manual bone preparation followed by an instrumented tibial trial. Soft-tissue release was directly titrated using real-time intraoperative pressure readings from the Verasense sensor.

Minor case-specific adjustments were made at the surgeon’s discretion. Exact numerical alignment targets and balancing thresholds were not available for retrospective reporting.

### Outcomes

The source dataset contained preoperative and postoperative knee range of motion (ROM, degrees); Knee Society Function and Knee scores (KSS-F and KSS-K, 0–100); and Oxford Knee Score (OKS, 0–48, higher scores better). Preoperative and postoperative pain was assessed using a 0 to 10 visual analogue scale (VAS), with 0 indicating no pain and 10 indicating the worst pain. Patients indicated their pain by marking a continuous line between these endpoints; the position of the mark was measured and recorded as the VAS score. KSS-K evaluates the operated knee, whereas KSS-F evaluates the patient’s walking and stair function [27]. Because identical KSS-F values had been duplicated across bilateral knee rows, KSS-F was analysed once per patient (Table S6). ROM, KSS-K, and OKS were retained as knee-level outcomes. VAS was stored at knee-record level.

Available postoperative outcomes were extracted from the final postoperative assessment block for each record. This represented the last available assessment rather than a prespecified common postoperative time point. Follow-up duration was documented for 158 of 173 knees. Detailed availability is reported in Online Resource 1, Table S2.

### Statistical analysis

No a priori sample-size calculation was performed because the study included all eligible records in a fixed retrospective dataset. The study was not designed as an equivalence or non-inferiority study, and no equivalence or non-inferiority margin was prespecified.

The analyses were reported in a transparent question-based sequence. Question 1 evaluated observed favourable improvement and was post hoc and clinically intuitive. Question 2 evaluated covariate-adjusted postoperative outcomes and was designated as the principal adjusted analysis. Question 3 evaluated baseline-severity effect modification and was post hoc and hypothesis-generating. All analyses used endpoint-specific complete cases without imputation.

For question 1, favourable improvement was defined for each observation as postoperative minus preoperative ROM, KSS-F, KSS-K, or OKS, and as preoperative minus postoperative VAS, so a positive value consistently represented improvement. For each endpoint, an ordinary least-squares model regressed improvement on technique group. A two-degree-of-freedom robust Wald test assessed the overall three-group difference. Holm correction controlled the five endpoint-level overall tests; the three pairwise contrasts per endpoint were reported with a separate Holm correction across 15 contrasts [28].

For question 2, the postoperative outcome was regressed on technique group, the corresponding preoperative outcome, age, sex, and BMI. Continuous covariates were standardised within each endpoint-specific analysis sample as (value minus sample mean) divided by sample standard deviation. Adjusted marginal means placed continuous covariates at their analysis-sample means and averaged predictions over the sample sex distribution. Overall two-degree-of-freedom Wald tests and pairwise contrasts used separate Holm families of five and 15 tests, respectively. Nominal two-sided 95% confidence intervals are shown; multiplicity decisions were based on Holm-adjusted P values.

ROM, VAS, KSS-K, and OKS used the knee as the observational unit. Type 1 cluster-robust covariance (CR1) estimates were clustered by patient so bilateral knees contributed to point estimation without being treated as statistically independent [29]. KSS-F used one observation per patient and type 1 heteroskedasticity-consistent (HC1) robust covariance. The planned pairwise contrasts were MAKO versus Conventional, Verasense versus Conventional, and Verasense versus MAKO.

For question 3, baseline severity was coded continuously so higher values indicated worse preoperative status, and postoperative outcomes were oriented so higher values indicated better status. Models included technique, baseline severity, their interaction, age, sex, and BMI. A two-degree-of-freedom robust Wald test assessed the two technique-by-severity terms, with Holm correction across five endpoints. Because interaction estimates can be driven by extrapolation when groups occupy different baseline ranges, models were repeated after restriction to the baseline interval represented in all three groups.

Sensitivity analyses used the simpler baseline-only adjusted model; excluded complex TKA or inferred-date records; retained one deterministic knee per patient for knee-level outcomes; and included diagnostic adjustments for calendar time or documented follow-up duration (Online Resource 1). Given the concentration of final VAS at zero, an exploratory pain-free analysis defined VAS = 0 and used a logistic model adjusted for baseline VAS, age, sex, and BMI with patient-clustered CR1 covariance; Holm correction covered its three pairwise contrasts.

Two post hoc common-window diagnostic analyses repeated the principal adjusted analysis among records with documented follow-up from 163 to 366 days, the observed range shared by all three groups, and from 180 to 365 days, an interval approximating 6 to 12 months within that shared range. The same covariates, analysis units, covariance estimators, complete-case rules, and Holm correction families were retained.

Descriptive summaries used mean ± standard deviation or median [interquartile range]. Baseline comparability was described using the maximum absolute pairwise standardised mean difference (SMD), not significance testing. Formal analyses were performed in R 4.6.1.

## Results

### Cohort and baseline characteristics

The locked cohort contained 173 knees from 139 patients: 36 Conventional, 92 MAKO, and 45 Verasense. Thirty-four patients contributed bilateral knees, and no bilateral patient crossed technique groups. The largest baseline imbalances were preoperative KSS-K (maximum |SMD| 0.55), BMI (0.42), sex (0.36), and preoperative VAS (0.33) (Table 1).

**Table 1:** Cohort characteristics and baseline outcomes by surgical technique

Characteristic	Conventional	MAKO	Verasense	Max |SMD|
Knees, n	36	92	45	—
Patients, n	30	74	35	—
Patients contributing two knees, n (%)	6/30 (20.0%)	18/74 (24.3%)	10/35 (28.6%)	0.20
Age, years, mean ± SD	70.8 ± 6.7	70.1 ± 6.1	69.2 ± 6.3	0.25
Female sex, n (%)	25/36 (69.4%)	65/92 (70.7%)	24/45 (53.3%)	0.36
Left knee, n (%)	19/36 (52.8%)	50/92 (54.3%)	26/45 (57.8%)	0.10
BMI, kg/m², mean ± SD	27.1 ± 4.2	28.9 ± 4.4	27.1 ± 5.4	0.42
Preoperative ROM, degrees	103.0 ± 11.4 (*n* = 35)	105.5 ± 15.3 (*n* = 92)	105.3 ± 14.5 (*n* = 45)	0.19
Preoperative VAS, points	7.3 ± 1.5 (*n* = 35)	7.3 ± 1.4 (*n* = 92)	7.7 ± 1.5 (*n* = 45)	0.33
Preoperative KSS-F, points—patients	51.6 ± 18.0 (*n* = 29)	51.8 ± 10.3 (*n* = 74)	51.1 ± 5.7 (*n* = 35)	0.07
Preoperative KSS-K, points	52.1 ± 9.4 (*n* = 35)	50.3 ± 13.8 (*n* = 92)	46.0 ± 12.5 (*n* = 45)	0.55
Preoperative OKS, points	22.4 ± 4.7 (*n* = 35)	23.3 ± 5.6 (*n* = 92)	22.4 ± 4.4 (*n* = 45)	0.17

Values are mean ± SD, median [IQR], or n (%). Except for patient counts, bilateral status, and KSS-F, values are summarised across knee records. KSS-F was counted once per patient. Max |SMD| denotes the largest absolute pairwise standardised mean difference and is not a P value. BMI, body mass index; ROM, range of motion; VAS, Visual Analogue Scale; KSS-F, Knee Society Function score; KSS-K, Knee Society Knee score; OKS, Oxford Knee Score. SD, standard deviation; IQR, interquartile range

Endpoint-specific paired analysis sets were 157 knees/125 patients for ROM, 149/120 for VAS, 141/113 for KSS-K, and 143/114 for OKS. KSS-F was analysed in 113 patients (26 Conventional, 59 MAKO, and 28 Verasense). Documented follow-up was available for 29/36 Conventional, 88/92 MAKO, and 41/45 Verasense knees. Median [IQR] follow-up was 365 [212 to 430], 398.5 [365.0 to 576.3], and 212 [180 to 288] days, respectively, with ranges of 142 to 1190, 34 to 750, and 163 to 366 days.

### Observed pre-to-postoperative improvement

Pain and functional scores improved substantially in all three groups, whereas mean ROM changed little. However, no endpoint showed a Holm-adjusted overall difference in favourable improvement among the techniques (0/5; Table 2, Panel A). VAS had the smallest nominal overall P value (*P* = 0.049), which became 0.247 after correction across the five endpoints.

None of the 15 pairwise improvement contrasts met the Holm-adjusted criterion (Table 2, Panel B). Verasense showed 0.769 points greater observed VAS improvement than MAKO (nominal 95% CI 0.152 to 1.386; raw *P* = 0.0146; Holm *P* = 0.2195). This was treated as a directional, hypothesis-generating signal rather than evidence of superiority.

**Table 2:** Observed favourable improvement by surgical technique

Panel A. Group means and robust overall tests						
Endpoint	Analysis *n* (C/M/V)	Conventional	MAKO	Verasense	Overall *P*	Holm *P* (5)
ROM	32/85/40 knees	−1.56 ± 12.73	−0.82 ± 15.21	−3.88 ± 15.95	0.694	1.000
VAS	32/79/38 knees	7.00 ± 1.70	6.81 ± 1.56	7.58 ± 1.46	0.049	0.247
KSS-F	26/59/28 patients	26.73 ± 20.78	24.41 ± 14.24	25.89 ± 13.75	0.821	1.000
KSS-K	31/74/36 knees	41.19 ± 9.33	41.28 ± 13.95	46.19 ± 13.24	0.202	0.808
OKS	31/76/36 knees	17.45 ± 5.50	15.67 ± 6.08	17.42 ± 5.32	0.280	0.841

Panel B. Pairwise differences in observed favourable improvement						
Endpoint	Contrast	Analysis *n*	Improvement difference	95% CI	Raw *P*	Holm *P* (15)
ROM	MAKO vs. Conventional	157 knees/125 patients	+ 0.739	−5.553 to 7.031	0.8180	1.0000
ROM	Verasense vs. Conventional	157/125	−2.313	−10.035 to 5.410	0.5572	1.0000
ROM	Verasense vs. MAKO	157/125	−3.051	−10.056 to 3.953	0.3932	1.0000
VAS	MAKO vs. Conventional	149/120	−0.190	−0.893 to 0.514	0.5968	1.0000
VAS	Verasense vs. Conventional	149/120	+ 0.579	−0.200 to 1.358	0.1452	1.0000
VAS	Verasense vs. MAKO	149/120	+ 0.769	0.152 to 1.386	0.0146	0.2195
KSS-F	MAKO vs. Conventional	113 patients	−2.324	−11.062 to 6.414	0.6022	1.0000
KSS-F	Verasense vs. Conventional	113 patients	−0.838	−10.257 to 8.581	0.8616	1.0000
KSS-F	Verasense vs. MAKO	113 patients	+ 1.486	−4.760 to 7.732	0.6410	1.0000
KSS-K	MAKO vs. Conventional	141 knees/113 patients	+ 0.090	−5.239 to 5.420	0.9735	1.0000
KSS-K	Verasense vs. Conventional	141/113	+ 5.001	−0.967 to 10.969	0.1005	1.0000
KSS-K	Verasense vs. MAKO	141/113	+ 4.911	−1.235 to 11.056	0.1173	1.0000
OKS	MAKO vs. Conventional	143 knees/114 patients	−1.781	−4.386 to 0.825	0.1804	1.0000
OKS	Verasense vs. Conventional	143/114	−0.035	−2.901 to 2.832	0.9809	1.0000
OKS	Verasense vs. MAKO	143/114	+ 1.746	−0.845 to 4.336	0.1866	1.0000

Values in Panel A are mean ± SD of individual favourable change scores computed within each endpoint-specific paired sample. Favourable improvement is postoperative minus preoperative for ROM, KSS-F, KSS-K, and OKS and preoperative minus postoperative for VAS; positive differences therefore indicate greater improvement in the first-named group. Knee-level models used patient-clustered CR1 covariance; KSS-F used one observation per patient and HC1 covariance. Panel A Holm P values control five endpoint-level overall tests. Panel B Holm P values separately control 15 pairwise contrasts. The 95% CIs are nominal and are not multiplicity-adjusted. No result met a Holm-adjusted 0.05 criterion. C, Conventional; M, MAKO; V, Verasense; CI, confidence interval; CR1, type 1 cluster-robust covariance; HC1, type 1 heteroskedasticity-consistent covariance; ROM, range of motion; VAS, Visual Analogue Scale; KSS-F, Knee Society Function Score; KSS-K, Knee Society Knee Score; OKS, Oxford Knee Score

### Adjusted postoperative outcomes

After adjustment for the corresponding baseline outcome, age, sex, and BMI, no endpoint showed a Holm-adjusted overall difference among the three techniques (0/5; Table 3, Panel A). The nominal overall VAS P value was 0.028 and became 0.141 after correction across five endpoints.

None of the 15 adjusted pairwise contrasts met the Holm-adjusted criterion (Table 3, Panel B). Verasense had an adjusted postoperative VAS 0.318 points lower than MAKO (nominal 95% CI − 0.579 to − 0.057; raw *P* = 0.0170; Holm *P* = 0.2546). Thus, the change-score and adjusted-postoperative analyses pointed in the same clinical direction for VAS, but neither constituted multiplicity-adjusted evidence.

**Table 3:** Covariate-adjusted postoperative outcomes by surgical technique

Panel A. Adjusted marginal means and robust overall tests						
Endpoint	Analysis *n* (C/M/V)	Conventional adjusted mean	MAKO adjusted mean	Verasense adjusted mean	Overall *P*	Holm *P* (5)
ROM	32/85/40 knees	102.39 (98.28–106.49)	104.56 (101.38–107.73)	101.66 (97.60–105.72)	0.495	1.000
VAS	32/79/38 knees	0.29 (− 0.03–0.61)	0.41 (0.18–0.64)	0.09 (− 0.03–0.21)	0.028	0.141
KSS-F	26/59/28 patients	78.41 (72.81–84.02)	77.17 (73.55–80.80)	75.82 (71.02–80.62)	0.811	1.000
KSS-K	31/74/36 knees	93.10 (91.50–94.71)	92.37 (90.84–93.91)	91.59 (90.18–92.99)	0.404	1.000
OKS	31/76/36 knees	40.16 (38.56–41.76)	39.09 (37.98–40.20)	39.56 (38.03–41.09)	0.546	1.000

Panel B. Adjusted pairwise postoperative outcome contrasts						
Endpoint	Contrast	Analysis *n*	Adjusted difference	95% CI	Raw *P*	Holm *P* (15)
ROM	MAKO vs. Conventional	157 knees/125 patients	+ 2.170	−3.170 to 7.510	0.4258	1.0000
ROM	Verasense vs. Conventional	157/125	−0.726	−6.647 to 5.195	0.8101	1.0000
ROM	Verasense vs. MAKO	157/125	−2.896	−8.020 to 2.228	0.2680	1.0000
VAS	MAKO vs. Conventional	149/120	+ 0.117	−0.293 to 0.527	0.5750	1.0000
VAS	Verasense vs. Conventional	149/120	−0.200	−0.520 to 0.119	0.2194	1.0000
VAS	Verasense vs. MAKO	149/120	−0.318	−0.579 to − 0.057	0.0170	0.2546
KSS-F	MAKO vs. Conventional	113 patients	−1.240	−7.727 to 5.247	0.7080	1.0000
KSS-F	Verasense vs. Conventional	113 patients	−2.595	−10.506 to 5.316	0.5203	1.0000
KSS-F	Verasense vs. MAKO	113 patients	−1.355	−7.355 to 4.644	0.6579	1.0000
KSS-K	MAKO vs. Conventional	141 knees/113 patients	−0.727	−2.852 to 1.398	0.5025	1.0000
KSS-K	Verasense vs. Conventional	141/113	−1.515	−3.720 to 0.691	0.1784	1.0000
KSS-K	Verasense vs. MAKO	141/113	−0.788	−3.031 to 1.456	0.4914	1.0000
OKS	MAKO vs. Conventional	143 knees/114 patients	−1.068	−2.994 to 0.859	0.2774	1.0000
OKS	Verasense vs. Conventional	143/114	−0.596	−2.859 to 1.668	0.6060	1.0000
OKS	Verasense vs. MAKO	143/114	+ 0.472	−1.418 to 2.362	0.6246	1.0000

Models included technique group, the corresponding standardised preoperative outcome, standardised age, sex, and standardised BMI. Differences are first-named minus second-named group on the original outcome scale; negative VAS differences and positive ROM/KSS/OKS differences favour the first-named group. Knee-level models used patient-clustered CR1 covariance; KSS-F used one observation per patient and HC1 covariance. Panel A Holm P values control five endpoint-level overall tests; Panel B values separately control 15 pairwise contrasts. The 95% CIs are nominal and are not multiplicity-adjusted. Model-based VAS confidence limits may extend slightly below zero. C, Conventional; M, MAKO; V, Verasense; BMI, body mass index; CI, confidence interval; CR1, type 1 cluster-robust covariance; HC1, type 1 heteroskedasticity-consistent covariance; ROM, range of motion; VAS, Visual Analogue Scale; KSS-F, Knee Society Function Score; KSS-K, Knee Society Knee Score; OKS, Oxford Knee Score

### Exploratory technique-by-baseline-severity interactions

No reliable technique-by-baseline-severity interaction was found for ROM, VAS, KSS-K, or OKS. KSS-F showed a full-sample interaction (Holm *P* = 0.000116), but baseline KSS-F ranges within the endpoint-specific paired interaction sample differed markedly across groups: 5–75 in Conventional, 30–70 in MAKO, and 45–60 in Verasense. Restricting the analysis to the shared 45–60 range reduced the sample from 113 to 98 patients and removed the interaction (Holm *P* = 0.4689). The available data therefore did not support selecting a technique according to preoperative severity (Table S5).

### VAS floor effect and sensitivity analyses

Final VAS was zero in 28/32 Conventional knees (87.5%), 62/79 MAKO knees (78.5%), and 35/38 Verasense knees (92.1%); overall, 125/149 analysed knees (83.9%) had no recorded postoperative pain. The post hoc pain-free logistic comparison was imprecise: for Verasense versus MAKO, the odds ratio was 3.62 (95% CI 0.68 to 19.16; Holm *P* = 0.392 across three contrasts). The continuous VAS signal therefore arose in a distribution with little remaining scale range to discriminate techniques. Sensitivity and diagnostic analyses did not alter the overall interpretation (Tables S3-S4).

Neither post hoc common-window diagnostic analysis yielded a Holm-adjusted overall or pairwise group difference (Table S7).

## Discussion

This study evaluated whether the three surgical approaches were associated with different postoperative clinical outcomes. We found no robust evidence of such differences. The observed magnitude of improvement did not reliably differ among techniques, and postoperative ROM, pain, and function did not reliably differ after adjustment for the corresponding baseline outcome, age, sex, and BMI. Preoperative severity did not show a reproducible technique-dependent association within baseline values represented in all three groups. These findings do not establish superiority, equivalence, or absence of technical benefit.

Compared with MAKO, Verasense showed 0.769 points greater observed VAS improvement and a 0.318-point lower adjusted postoperative VAS. Neither contrast met its Holm-adjusted criterion. These are related analyses of the same observations, not independent replications. Danoff et al. reported an inpatient post-TKA VAS MCID of 22.6 mm, equivalent to 2.26 points on a 0 to 10 scale [30]. Both estimates in the present cohort were smaller. This comparison provides clinical context rather than a formal MCID assessment because the published threshold concerns within-patient change during hospitalisation, whereas our estimates are between-group differences at later and nonuniform follow-up. The observed pattern therefore remains hypothesis-generating and does not establish a clinically important pain benefit.

The ROAM randomised trial evaluated a combined robotic-arm and pressure-sensor strategy rather than either technology in isolation. It reported greater improvement in WOMAC pain in a longitudinal analysis of assessments at 2, 6, and 12 months after surgery, without a corresponding functional advantage [31]. This finding provides context for the directional VAS pattern in our cohort, although the combined intervention cannot identify the contribution of either component. No combined robotic-plus-sensor group was available in the present cohort.

The technical and clinical outcomes answer different but related questions. MAKO provides geometry-based planning and information on alignment, component position, and joint-gap distances, whereas Verasense provides compartment-load feedback to guide soft-tissue balancing [9]. Prior studies suggest that robotic-arm assistance can improve the accuracy and repeatability of planned bone preparation [4, 6–8, 22–25], while pressure-sensor guidance can quantify compartment loads and may improve balance in selected settings [10–17]. Improvements in these technical targets may increase operative consistency or reduce technical outliers without necessarily producing a detectable short-term difference in pain, ROM, or function. Conversely, the absence of a clear postoperative group difference does not establish that alignment accuracy, component position, gap balance, or compartment loads were similar across the approaches. Geometry and compartment load may also represent complementary aspects of knee balance rather than competing measures [7, 9, 11]. A complete evaluation should therefore assess whether each approach achieves its intended technical target and whether target attainment is associated with subsequent clinical recovery.

MAKO and Verasense are surgeon-directed forms of digital intraoperative support. Recent perspectives describe how digital surgery may develop further through AI-assisted decision support, greater automation, and remote surgical workflows [32, 33].

Technology selection should not be based on these outcome comparisons alone. Surgeon expertise, anatomy, alignment philosophy, equipment availability, workflow, cost, and technology-specific risks remain relevant. The exploratory severity analysis did not support a rule that patients with worse baseline scores should receive one particular technique.

Several limitations constrain interpretation. The study was retrospective and nonrandomised, and technique was closely associated with calendar period. In particular, the observed MAKO and Verasense procedure periods did not overlap, so their comparison could not separate surgical approach from changes in clinical practice or experience over time. Although all three surgeons contributed cases to every group, case-linked surgeon identifiers, exact case counts by surgeon, and individual reasons for technique selection were unavailable. Surgeon preference, surgeon-specific case mix, patient selection, and learning effects therefore remain possible sources of residual confounding.

The final postoperative assessment was not obtained at a prespecified common interval, and median documented follow-up was shortest in the Verasense group. Because ROM, VAS, KSS, and OKS may change during recovery, the between-group estimates may partly reflect differences in assessment timing. Neither post hoc common-window diagnostic analysis produced a Holm-adjusted overall or pairwise group difference. These analyses reduced variation in follow-up timing but used smaller and imbalanced samples and cannot eliminate incomplete follow-up, selection into the restricted windows, or confounding by the nonconcurrent treatment periods.

Unequal group sizes and endpoint-specific attrition reduced precision. Because no single primary endpoint had been prospectively designated, Holm correction was applied across the stated test families, raising the evidentiary threshold for individual contrasts. Several nominal 95% confidence intervals remained wide. The absence of Holm-adjusted differences therefore neither excludes clinically important effects nor establishes equivalence.

The study did not capture case-level achieved robotic alignment accuracy, component position, joint-gap measurements, final Verasense compartment loads, or the number and type of intraoperative balancing adjustments. It could therefore not test the complete proposed pathway from technology-specific guidance, through achievement of the intended intraoperative target, to postoperative recovery. In particular, the analyses could not determine whether the intended technical objectives were achieved or whether target attainment was associated with postoperative pain, ROM, or function. The largely negative clinical comparisons should therefore be interpreted as comparisons of recorded postoperative outcomes among the three surgical groups, rather than as an evaluation of the technical pathway linking intraoperative guidance to recovery. The absence of verified numerical alignment targets and balancing thresholds limits precise replication of the operative protocols and prevents assessment of procedural variation within each technique. The MAKO group used restricted kinematic alignment, whereas the Conventional and Verasense groups used mechanical alignment. The contribution of the technology could therefore not be separated from that of the accompanying alignment strategy.

Operative-time data were incomplete and unevenly available across the groups, and cost data were unavailable. Future prospective comparisons should collect technical, clinical, workflow, and cost outcomes together at prespecified time points.

The final postoperative VAS was zero in 125 of 149 analysed knees, producing a marked floor effect. Although this may partly reflect genuine pain relief, it leaves little remaining range with which to distinguish between techniques. Replacing VAS with another single pain-intensity scale would not necessarily remove this problem [34]. Future knee-level studies should combine side-specific KOOS Pain with separate pain ratings at rest and during a standardised activity, collected at common prespecified postoperative times [34, 35]. FJS-12 at 12 months could add information on joint awareness among otherwise well-recovered patients, but it would complement rather than replace a pain-specific outcome [36].

In this retrospective cohort of 173 CR-TKAs in 139 patients, no comparison of observed improvement or covariate-adjusted postoperative ROM, VAS, or function met the multiplicity-adjusted criterion for a group difference among Conventional, MAKO, and Verasense. Preoperative severity did not show a reproducible technique-dependent association within baseline ranges represented across all three groups. A small directional Verasense-versus-MAKO VAS pattern appeared in both analyses but did not meet the multiplicity-adjusted criterion and remains hypothesis-generating. These findings do not establish equivalence. They define a focused pain hypothesis for prospective evaluation at common follow-up intervals using more discriminating pain measures and linked technical outcomes.

## Supplementary Information

Below is the link to the electronic supplementary material.

Supplementary Material 1: Supplementary methods and Tables S1-S7, including endpoint availability, follow-up, common-window diagnostic analyses, sensitivity analyses, the Visual Analogue Scale floor effect, baseline-severity effect modification, and the patient-level Knee Society Function Score audit

## Data Availability

The de-identified analytical dataset may be available from the corresponding author on reasonable request, subject to institutional and ethics approval and applicable data-protection requirements. The identifiable source dataset is not publicly available because it contains protected clinical information.

## Code availability

The complete base-R reproducibility code is maintained in the project audit workspace and is available from the corresponding author on reasonable request. No persistent public repository identifier was assigned at submission.
