# Supplementary material for: Postoperative outcomes after conventional, robotic-arm-assisted, and pressure-sensor-guided cruciate-retaining total knee arthroplasty: a retrospective cohort study
Source: J Robot Surg. 2026 Sep 15;20(1):988. doi: 10.1007/s11701-026-03954-w (PMC13578005; doi:10.1007/s11701-026-03954-w)
Supplement: Supplementary file 1 [file 11701_2026_3954_MOESM1_ESM.pdf]

Supplementary methods and Tables S1–S7

Article title: Postoperative Outcomes After Conventional, Robotic-Arm-Assisted, and Pressure-Sensor-Guided Cruciate-Retaining Total Knee Arthroplasty: A Retrospective Cohort Study

Abbreviations: BMI, body mass index; CI, confidence interval; CR1, type 1 cluster-robust covariance; HC1, type 1 heteroskedasticity-consistent covariance; KSS-F, Knee Society Function Score; KSS-K, Knee Society Knee Score; OKS, Oxford Knee Score; OR, odds ratio; ROM, range of motion; SMD, standardised mean difference; TKA, total knee arthroplasty.

The principal adjusted analysis is the covariate-adjusted postoperative comparison. Observed favourable improvement and baseline-severity effect modification are post hoc exploratory analyses. Multiplicity is controlled separately within each stated family.

Supplementary Methods

The cohort contained 173 knees in 139 patients. ROM, recorded 0–10 pain score, KSS-K, and OKS were analysed at knee level using type 1 cluster-robust covariance (CR1), clustered by patient. KSS-F was analysed once per patient using type 1 heteroskedasticity-consistent covariance (HC1) because the same walking-and-stair function score was duplicated across bilateral knee rows. Endpoint-specific paired complete cases were used without imputation.

Observed favourable improvement was analysed as change ~ technique; the principal adjusted model was postoperative outcome ~ technique + corresponding baseline outcome + age + sex + BMI. Baseline-severity models added continuous severity and technique-by-severity interaction terms. Five endpoint-level robust Wald tests and 15 pairwise contrasts were corrected as separate Holm families. Severity interactions were corrected across five endpoint-level tests and repeated within the common baseline range represented in all three groups.

Sensitivity specifications used the baseline-only model; excluded complex TKA records; excluded records with inferred surgery dates; retained one deterministic knee per patient; or included diagnostic adjustments for calendar time or documented follow-up duration. Two additional post hoc diagnostics restricted documented follow-up to 163 to 366 days and 180 to 365 days while retaining the principal covariate set and Holm families. A zero-score analysis modelled whether the final recorded pain score was 0 using logistic regression adjusted for baseline pain score, age, sex, and BMI with patient-clustered CR1 covariance.

Supplementary Table S1. Endpoint availability and missing postoperative outcomes

Outcome	Group	Eligible unit n	Baseline n	Final n	Paired n	Final missing n (%)
ROM	Conventional	36 knees	35	32	32	4 (11.1%)
ROM	MAKO	92 knees	92	85	85	7 (7.6%)
ROM	Verasense	45 knees	45	40	40	5 (11.1%)
Pain score	Conventional	36 knees	35	32	32	4 (11.1%)
Pain score	MAKO	92 knees	92	79	79	13 (14.1%)
Pain score	Verasense	45 knees	45	38	38	7 (15.6%)
KSS-F	Conventional	30 patients	29	26	26	4 (13.3%)
KSS-F	MAKO	74 patients	74	59	59	15 (20.3%)
KSS-F	Verasense	35 patients	35	28	28	7 (20.0%)
KSS-K	Conventional	36 knees	35	31	31	5 (13.9%)
KSS-K	MAKO	92 knees	92	74	74	18 (19.6%)
KSS-K	Verasense	45 knees	45	36	36	9 (20.0%)
OKS	Conventional	36 knees	35	31	31	5 (13.9%)
OKS	MAKO	92 knees	92	76	76	16 (17.4%)
OKS	Verasense	45 knees	45	36	36	9 (20.0%)

KSS-F denominators are patients; all other denominators are knees. Paired n requires both the corresponding preoperative and final value. No outcome was imputed.

Supplementary Table S2. Documented follow-up by technique group

Group	Locked knees	Recorded n (%)	≥365 days n (%)	Mean ± SD, days	Median [IQR], days	Range, days
Conventional	36	29 (80.6%)	15 (41.7%)	433.6 ± 307.9	365 [212–430]	142–1190
MAKO	92	88 (95.7%)	67 (72.8%)	467.0 ± 150.5	398.5 [365.0–576.3]	34–750
Verasense	45	41 (91.1%)	2 (4.4%)	234.9 ± 69.1	212 [180–288]	163–366

Percentages use all locked knees in each group. Follow-up duration was not documented for every knee and did not represent a common prespecified postoperative assessment interval.

Supplementary Table S3. Robustness of the Verasense-versus-MAKO adjusted postoperative pain-score contrast

Role	Specification	Knees/patients	Adjusted difference	95% CI	Raw P	Holm P (15)	Positive contrasts/15
Principal	Baseline + age + sex + BMI	149/120	−0.318	−0.579 to −0.057	0.0170	0.2546	0
Sensitivity	Baseline only	149/120	−0.366	−0.634 to −0.098	0.0074	0.1115	0
Sensitivity	Exclude complex TKA	142/115	−0.349	−0.628 to −0.070	0.0142	0.2137	0
Sensitivity	Exclude inferred dates	146/117	−0.325	−0.588 to −0.062	0.0153	0.2293	0
Sensitivity	One knee per patient	120/120	−0.402	−0.692 to −0.112	0.0066	0.0987	0
Diagnostic	+ calendar time	149/120	−0.481	−0.796 to −0.166	0.0028	0.0414	1
Diagnostic	+ documented follow-up	143/115	−0.475	−0.794 to −0.156	0.0035	0.0527	0

Negative values indicate lower postoperative pain in Verasense. Each specification has its own Holm correction across 15 contrasts; the six sensitivity/diagnostic specifications are not one 90-test family. Calendar-time and follow-up results are diagnostic because technique was strongly aligned with period and documented follow-up timing was incomplete and nonuniform. The pairwise calendar-time result crossed its Holm threshold, whereas the corresponding five-outcome overall test did not; it cannot identify technique independently of period.

Supplementary Table S4. Pain-score floor effect and binary zero-score diagnostic

Panel A. Final pain score=0 by group

Group	Analysed knees	Pain score=0, n	Pain score=0, %
Conventional	32	28	87.5
MAKO	79	62	78.5
Verasense	38	35	92.1

Panel B. Pairwise odds ratios for a final recorded score of 0

Contrast	Knees/patients	Odds ratio	95% CI	Raw P	Holm P (3)
MAKO vs Conventional	149/120	0.46	0.12 to 1.83	0.272	0.544
Verasense vs Conventional	149/120	1.67	0.27 to 10.52	0.584	0.584
Verasense vs MAKO	149/120	3.62	0.68 to 19.16	0.131	0.392

The binary model adjusted for baseline pain score, age, sex, and BMI and used patient-clustered CR1 covariance. Wide confidence intervals and the absence of a Holm-adjusted difference do not support a superiority claim.

Supplementary Table S5. Post hoc baseline-severity effect-modification analyses

Endpoint	Full n	Common baseline range	Full interaction P	Full Holm P (5)	Common-support n	Common-support interaction P	Common-support Holm P (5)
ROM	157 knees/125 patients	70–125	0.3568	1.0000	153 knees/121 patients	0.6127	0.9279
Pain score	149/120	4–9	0.6747	1.0000	144/117	0.4108	0.9279
KSS-F	113 patients	45–60	<0.0001	0.000116	98 patients	0.0938	0.4689
KSS-K	141/113	35–66	0.3662	1.0000	117/95	0.1214	0.4857
OKS	143/114	15–34	0.8028	1.0000	135/108	0.3093	0.9279

KSS-F baseline support by group

Group	Full range	Patients in full model	Patients in common range 45–60
Conventional	5–75	26	19
MAKO	30–70	59	51
Verasense	45–60	28	28

Models included technique, continuously coded baseline severity, technique-by-severity terms, age, sex, and BMI. The 2-df interaction test was Holm-corrected across five endpoints. The KSS-F full-sample signal did not persist after restriction to the range observed in all three groups; no technique-selection rule is supported.

Supplementary Table S6. Patient-level KSS-F audit and paired descriptives

Group	Baseline available, n	Baseline mean ± SD	Paired n	Paired preoperative	Paired postoperative	Favourable improvement
Conventional	29	51.6 ± 18.0	26	52.31 ± 14.02	79.04 ± 14.28	26.73 ± 20.78
MAKO	74	51.8 ± 10.3	59	52.29 ± 8.06	76.69 ± 16.98	24.41 ± 14.24
Verasense	35	51.1 ± 5.7	28	50.36 ± 5.76	76.25 ± 14.51	25.89 ± 13.75

Supplementary Table S7. Post hoc common-window diagnostic analyses

Panel A. Robust overall three-group tests

Window	Endpoint	C/M/V n	Total n	Wald χ²	Raw P	Holm P (5)
163–366 days	ROM	13/19/40	72 observations/57 patients	1.644	0.4396	1.0000
163–366 days	Pain score	13/17/38	68 observations/55 patients	3.096	0.2127	0.8508
163–366 days	KSS-F	10/13/28	51 observations/51 patients	0.297	0.8620	1.0000
163–366 days	KSS-K	12/17/36	65 observations/52 patients	5.045	0.0803	0.4013
163–366 days	OKS	12/17/36	65 observations/52 patients	0.702	0.7041	1.0000
180–365 days	ROM	11/18/28	57 observations/45 patients	2.730	0.2554	0.7662
180–365 days	Pain score	11/16/26	53 observations/43 patients	5.295	0.0708	0.3541
180–365 days	KSS-F	8/12/20	40 observations/40 patients	0.612	0.7362	1.0000
180–365 days	KSS-K	10/16/25	51 observations/41 patients	3.924	0.1406	0.5622
180–365 days	OKS	10/16/25	51 observations/41 patients	1.245	0.5365	1.0000

Panel B. Pairwise adjusted postoperative outcome contrasts

Panel B1: 163–366 days

Endpoint	Contrast	n/patients	Difference	95% CI	Raw P	Holm P (15)
ROM	MAKO vs Conventional	72/57	4.230	-5.500 to 13.960	0.3941	1.0000
ROM	Verasense vs Conventional	72/57	-0.324	-8.493 to 7.845	0.9380	1.0000
ROM	Verasense vs MAKO	72/57	-4.554	-11.572 to 2.464	0.2034	1.0000
Pain score	MAKO vs Conventional	68/55	0.291	-0.482 to 1.064	0.4609	1.0000
Pain score	Verasense vs Conventional	68/55	-0.171	-0.555 to 0.212	0.3810	1.0000
Pain score	Verasense vs MAKO	68/55	-0.462	-1.096 to 0.172	0.1528	1.0000
KSS-F	MAKO vs Conventional	51/51	-3.138	-15.369 to 9.093	0.6151	1.0000
KSS-F	Verasense vs Conventional	51/51	-0.484	-10.325 to 9.356	0.9231	1.0000
KSS-F	Verasense vs MAKO	51/51	2.653	-8.102 to 13.409	0.6287	1.0000
KSS-K	MAKO vs Conventional	65/52	2.784	-1.203 to 6.771	0.1712	1.0000
KSS-K	Verasense vs Conventional	65/52	-0.384	-3.938 to 3.170	0.8322	1.0000

Endpoint	Contrast	n/patients	Difference	95% CI	Raw P	Holm P (15)
KSS-K	Verasense vs MAKO	65/52	-3.168	-5.958 to -0.378	0.0260	0.3907
OKS	MAKO vs Conventional	65/52	-1.244	-4.904 to 2.415	0.5052	1.0000
OKS	Verasense vs Conventional	65/52	-0.085	-3.076 to 2.906	0.9556	1.0000
OKS	Verasense vs MAKO	65/52	1.159	-1.660 to 3.978	0.4202	1.0000

Panel B2: 180–365 days (6 months to 12 months post-operation)

Endpoint	Contrast	n/patients	Difference	95% CI	Raw P	Holm P (15)
ROM	MAKO vs Conventional	57/45	7.614	-2.332 to 17.561	0.1335	1.0000
ROM	Verasense vs Conventional	57/45	6.340	-2.059 to 14.739	0.1390	1.0000
ROM	Verasense vs MAKO	57/45	-1.275	-9.322 to 6.773	0.7562	1.0000
Pain score	MAKO vs Conventional	53/43	0.258	-0.587 to 1.103	0.5498	1.0000
Pain score	Verasense vs Conventional	53/43	-0.268	-0.719 to 0.183	0.2437	1.0000
Pain score	Verasense vs MAKO	53/43	-0.526	-1.145 to 0.093	0.0957	1.0000
KSS-F	MAKO vs Conventional	40/40	-4.437	-18.711 to 9.837	0.5423	1.0000
KSS-F	Verasense vs Conventional	40/40	-0.026	-12.514 to 12.463	0.9968	1.0000
KSS-F	Verasense vs MAKO	40/40	4.411	-7.187 to 16.010	0.4560	1.0000
KSS-K	MAKO vs Conventional	51/41	3.551	-0.910 to 8.012	0.1187	1.0000
KSS-K	Verasense vs Conventional	51/41	0.565	-3.735 to 4.865	0.7968	1.0000
KSS-K	Verasense vs MAKO	51/41	-2.986	-6.306 to 0.334	0.0779	1.0000
OKS	MAKO vs Conventional	51/41	-1.421	-5.607 to 2.765	0.5058	1.0000
OKS	Verasense vs Conventional	51/41	0.292	-3.531 to 4.115	0.8811	1.0000
OKS	Verasense vs MAKO	51/41	1.713	-1.326 to 4.751	0.2692	1.0000

Two post hoc diagnostic analyses restricted records to the intersection of the observed documented follow-up ranges across all three groups (163 to 366 days) and to a clinically interpretable interval approximating 6 to 12 months after surgery (180 to 365 days). Both restrictions were post hoc diagnostics and did not alter the main analysis hierarchy. Models adjusted for the corresponding baseline outcome, age, sex, and BMI. Knee-level outcomes used patient-clustered CR1 covariance; KSS-F used one observation per patient with HC1 covariance. Holm correction was applied separately across five overall endpoint tests and 15 pairwise contrasts within each window. No overall or pairwise comparison met its Holm-adjusted 0.05 criterion. Confidence intervals are nominal. C, Conventional; M, MAKO; V, Verasense.
